# Supplementary material for: Exploiting the Features of Short Peptides to Recognize Specific Cell Surface Markers
Source: Int J Mol Sci. 2023 Oct 26;24(21):15610. doi: 10.3390/ijms242115610 (PMC10650159; doi:10.3390/ijms242115610)
Supplement: Supplementary file 1 [file ijms-24-15610-s001.zip › ijms-2661876-supplementary.pdf]

International Journal of *Molecular Sciences*

MDPI

Supporting information

Exploiting the Features of Short Peptides to Recognize Specific Cell Surface Markers

Michela Buonocore [1,2], Manuela Grimaldi [1], Angelo Santoro [1,3], Verdiana Covelli [1,4], Carmen Marino [1,5], Enza Napolitano [1,5], Sara Novi [1,5], Mario Felice Tecce [1], Elena Ciaglia [6], Francesco Montella [6], Valentina Lopardo [6], Valeria Perugini [7], Matteo Santin [7] and Anna Maria D'Ursi [1,*]

[1] Department of Pharmacy, University of Salerno, 84084 Fisciano, Italy; mbuonocore@unisa.it or michela.buonocore@unina.it (M.B.); magrimaldi@unisa.it (M.G.); asantoro@unisa.it (A.S.); vcovelli@unisa.it or verdiana.covelli@unina.it (V.C.); cmarino@unisa.it (C.M.); enapolitano@unisa.it (E.N.); snovi@unisa.it (S.N.); tecce@unisa.it (M.F.T.)
[2] Department of Chemical Sciences, University of Naples Federico II, 80138 Naples, Italy
[3] Scuola di Specializzazione in Farmacia Ospedaliera, University of Salerno, 84084 Fisciano, Italy
[4] Department of Pharmacy, School of Medicine and Surgery, University of Naples Federico II, 80138 Naples, Italy
[5] PhD Program in Drug Discovery and Development, Department of Pharmacy, University of Salerno, 84084 Fisciano, Italy
[6] Department of Medicine, Surgery and Dentistry "Scuola Medica Salernitana", University of Salerno, 84081 Baronissi, Italy; eciaglia@unisa.it (E.C.), fmontella@unisa.it (F.M.), vlopardo@unisa.it (V.L.)
[7] Centre for Regenerative Medicine and Devices, School of Applied Sciences, University of Brighton, Brighton BN2 4AT, UK; v.perugini@brighton.ac.uk (V.P.); m.santin@brighton.ac.uk (M.S.)
* Correspondence: dursi@unisa.it

S1: Western Blot

A Western Blot assay was performed to verify CD44 expression on MSCs compared to the human hepatocarcinoma (HepG2) cell line. The anti-GAPDH, an ubiquitary protein, was used as the loading control. From the blots shown in **Figure S1a**, anti-CD44 shows to react with the markers on the hBM-MSCs and not on the HepG2. Furthermore, the detection of the GAPDH made possible the quantification of these expressions by calculating the ratio of markers blot *vs* GAPDH blot (**Figure S1b**), which confirmed the expression of CD44 on the cellular surface.

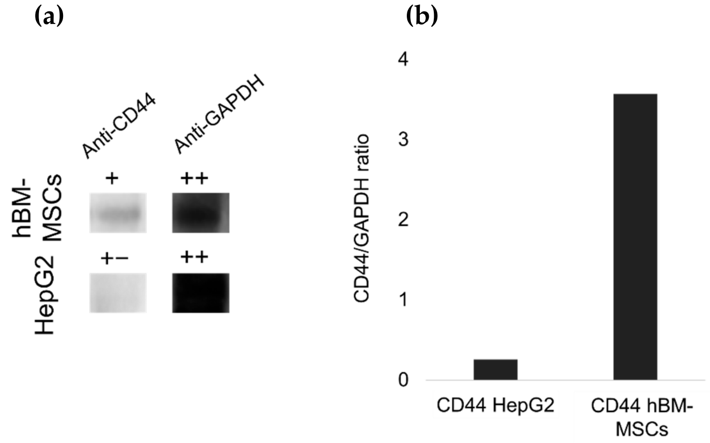

Figure S1. a) Expression of CD44 and GAPDH (control) of hBM-MSCs compared to HepG2 cell line. **b)** Quantification of CD44 in HepG2 and hBM-MSCs with GAPDH expression.

S2: Viability assay

The colorimetric viability assay was performed using CCK-8 kit, containing WST-8, to evaluate hBM-MSCs cell viability after exposition to DTYCF and PNHSE at different concentrations (0.1, 0.4, 1.5, 6, 25 and 100 μM). In view of the potential rapid "diagnostic" application of these peptides, we measured the toxicity at 24 h. After exposure to the different concentrations of peptides, analysis of cell viability after 24 h shows that cell viability is not affected by the peptides even at the highest concentration (100 μM) (**Figure S2**).

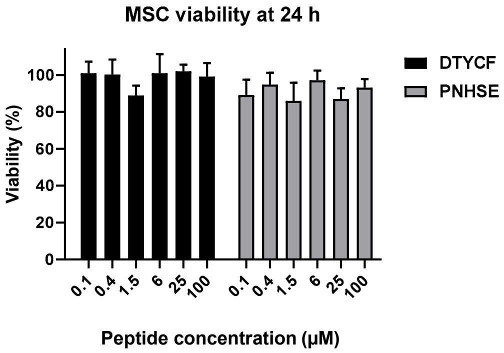

Figure S2. Histogram graph showing the percentage of the cell viability at 24 h after the exposition of different concentrations of peptides.
